# Morbidity and mortality among infants of diabetic mothers admitted to a neonatal care unit in Karbala pediatric teaching hospital

**DOI:** 10.25122/jml-2022-0073

**Published:** 2022-08

**Authors:** Moatasem Ghazi Hasoon Almhanna, Zuhair Omran Easa, Sabah Hassan Ali Alatwani

**Affiliations:** 1 Department of Pediatrics, College of Medicine, University of Karbala, Karbala, Iraq

**Keywords:** infant, diabetic, macrosomia, hypoglycemia

## Abstract

This study aimed to determine the morbidity and mortality patterns among infants of diabetic mothers admitted to the neonatal care unit in Karbala pediatric teaching hospital. The study enrolled fifty diabetic infants (pregestational and gestational) admitted to the ward from the 1^st^ of October 2013 to the 30^th^ of January 2014. Data on delivery mode, gestational age, birth weight, other associated morbidities, investigation results, therapy, length of hospital stay, and outcome were collected and compared to infants of non-diabetic mothers admitted during the same period. A retrospective analysis of maternal data was performed. 62% of infants were born to mothers with gestational diabetes, and 38% were born to mothers with pre-gestational diabetes. 86% were born by caesarian section, of which 35% were by emergency cesarean section. The mean gestational age of infants of diabetic mothers was 37w1d±1.88, and 29 (64%) had macrosomia. The most typical morbidities were hypoglycemia (significantly higher in infants of diabetic mothers (IDMs) than infants of non-diabetic mothers) and hyperbilirubinemia in 36 (72%) and 24 (48%), respectively. There was no difference in morbidity patterns between infants of pregestational and gestational diabetic mothers except for macrosomia, and transient tachypnea of newborns was higher in gestational diabetes. The mortality rate was not significantly higher in IDMs. Diabetes during pregnancy has a serious effect on neonates and their mothers. The commenced morbidities in IDMs were hypoglycemia, macrosomia, and hyperbilirubinemia, so strict control of blood glucose level during pregnancy and education of diabetic women is essential before and during gestation.

## INTRODUCTION

Diabetes is a major medical issue associated with pregnancy. Pre-existing diabetes mellitus is thought to complicate 0.2% to 0.3% of all pregnancies, with another 1–5% having gestational diabetes mellitus [1]. Pregestational diabetes is becoming more common and is linked to an increased risk of congenital abnormalities, macrosomia, preeclampsia, and premature birth. Perinatal mortality rate (PMR) in people with diabetes mellitus varies greatly, with congenital abnormalities and preterm labor being the main causes of greater PMR. Women with gestational diabetes mellitus or poor glucose tolerance are a diverse group with a range of PMRs, especially if insulin is required during pregnancy [2]. Respiratory distress, growth limits, polycythemia, hypoglycemia, congenital abnormalities, hypocalcemia, and hypomagnesemia are all common morbidities in diabetic infants [3]. Macrosomia is caused by increased insulin production affecting an infant's growth while still in the womb. Infants are born with more adipose tissue or fat, more glycogen in their livers, and larger organs (*e.g*., the heart) [4]. Congenital abnormalities affect roughly 4–12% of babies born to type 1 diabetic mothers (there is no increased incidence among gestational diabetic mothers) [5]. One of the most prevalent malformations linked with insulin-dependent diabetes during pregnancy is caudal agenesis. Ventricular septal defect and other heart abnormalities, neural tube defects, small left colon syndrome, and genitourinary abnormalities have also been reported [6]. Fetal and infant mortality and morbidity rates among IDMs have improved due to fetal surveillance, perinatal care, and high-level neonatal care. Prior to or throughout early pregnancy, women with poorly controlled type 1 diabetes had a 4–10% chance of having a significant birth defect and a 15–20% chance of spontaneous abortion [7, 8].

## Material and Methods

A hundred patients were enrolled in our study with an age range of one hour to twenty-six days, admitted to Karbala teaching hospital for children nursery unit from the 1^st^ of October 2013 to the 30^th^ of January 2014. Fifty patients were IDM, and for each IDM, we enrolled infants of healthy (no diabetes mellitus, no chronic disease) women admitted to the unit within the same period who matched gestational age as control. Full history was taken from the mother regarding age, gravida, parity, type of diabetes mellitus (DM), previous birth (stillbirth, hypoglycemia, congenital anomalies), antenatal care, presence of associated disease, drug history for the treatment of DM or other diseases. The patients were assessed for the type of delivery (normal vaginal delivery (NVD) and cesarean section), gestational age (by physical examination, history, and ultrasound), body weight, signs of birth trauma, respiratory distress, hypoglycemia, hypocalcemia, polycythemia, cardiac and gross congenital anomalies. Routine investigations were carried out, including blood sugar (done for all neonates (by heel prick and read by reflectometer, in the first hour of admission and repeated hourly for the first 4–6 hrs., then every 4 hr. Serum calcium level was measured for all patients, even with normal blood sugar, and samples were aspirated from the peripheral vein without a tourniquet. Packed-cell volume (PCV): done for all patients, samples aspirated from the central vein. Total serum bilirubin: done for all patients, by heel prick and a read by the bilirubin meter, on the first day of admission, and follow up were done according to clinical examination.

Non-routine investigations included:

- Chest X-ray (CXR): for patients with signs of respiratory distress;
- Echo study: for patients suspected to have cardiac anomalies;
- Blood culture: for patients suspected to have sepsis;
- HbAlc was done for each diabetic mother.

### Statistical Analysis

For statistical analysis, data were entered into an Excel spreadsheet (Microsoft Corp, Redmond, WA) and imported into SPSS version 16.0 software (SPSS Inc, Chicago, IL).

## Results

**Figure 1:**
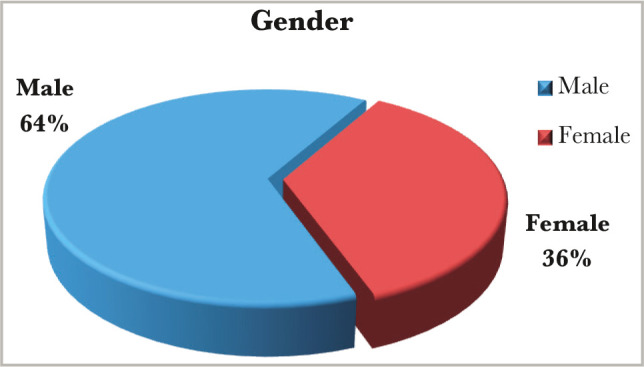
Gender distribution in the study group.

**Figure 2:**
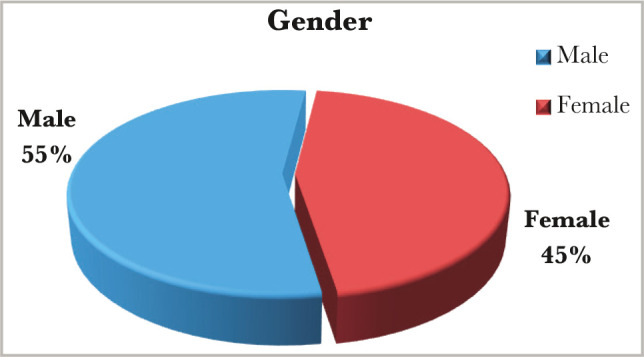
Gender distribution in the control group.

A total of a hundred patients, fifty of them born to diabetic mothers with a mean age of 2.52-day (range from 1h to 26 days) study group, and fifty patients born to healthy mothers (no diabetic, no chronic disease) with a mean age of 2.35-day (range from 1 hr. to 8 days) control group, enrolled in our study from the 1^st^ of October 2013 to the 30^th^ of January 2014. In our study, there was a slight male predominance. In the study group male to female ratio was 1.8:1, while in the control group male to female ratio was 1.2:1, as shown in Figures 1 and 2.

**Figure 3:**
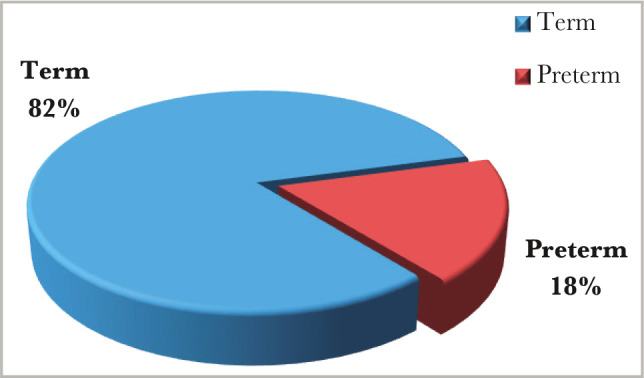
Term to preterm ratio in study and control groups.

Regarding the gestational age (GA), the percentage of term and preterm patients were similar for the study and control groups, (n=41) 82% term and (n=9) 18% preterm, with the mean gestational age for the study group of 37 weeks (range from 33 w2d_38 w3d), and mean gestational age for the control group of 37 weeks (range from 33 w5d_38 weeks) as shown in Figure 3.

**Figure 4:**
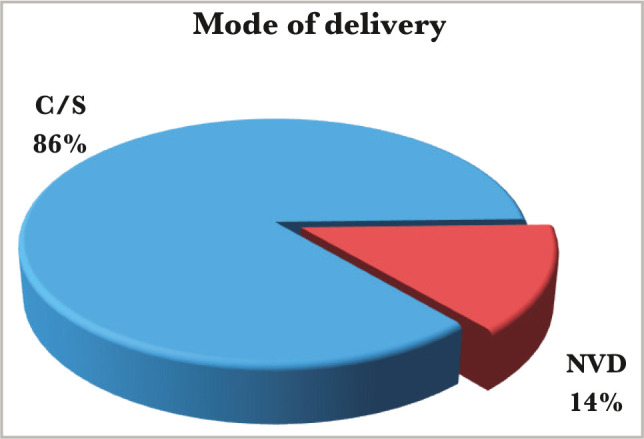
Mode of delivery of the study group.

**Figure 5:**
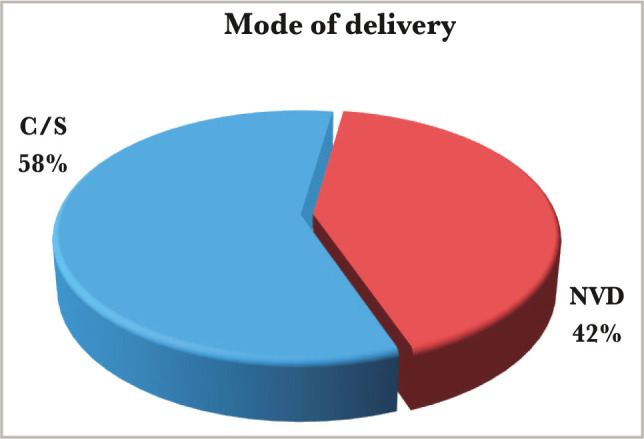
Mode of delivery of the control group.

Among the study group, the weight of term patients ranged from 2.5 kg to 5.2 kg, with an average of 4.12 kg. 32 cases (64%) were macrosomia, and the weight of preterm patients for the same group ranged from 2 kg to 3.1 kg, with an average of 2.6 kg. In the control group, the weight of term patients ranged from 2.3 kg to 4.4 kg with an average of 3.8 kg. 6 cases (12%) were macrosomia, and the weight of the preterm patients ranged from 1.9 kg to 2.25 kg with an average of 2.14 kg. The type of labor showed a wide difference between the study and the control group. In the study group, there were 86% (n=43) of cases with elective C/S, higher than emergency C/S (65% elective versus 35% emergency), while NVD was (n=7) 14%. In the control group, there were 58% (n=29) of cases C/S, and 42% (n=21) of NVD (Figures 4 and 5).

**Figure 6:**
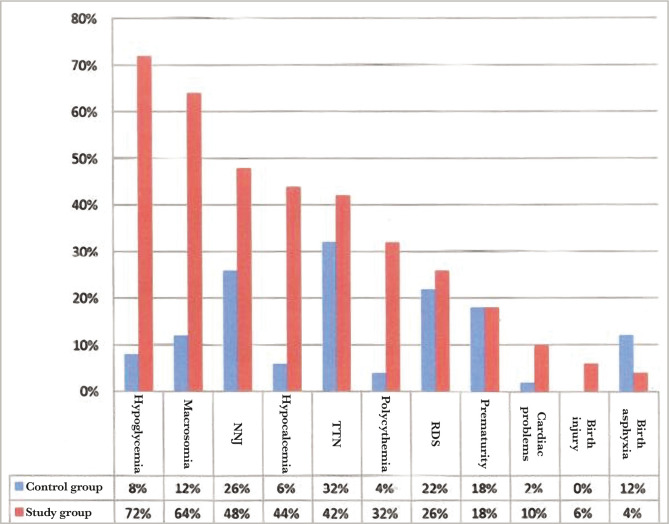
Morbidity comparison between study and control.

Two infants died in the study group, so the mortality rate was 4%. The cause of death for the first case was a preterm birth with cardiomyopathy complicated by heart failure and respiratory distress. In the second case, a full-term baby weighing 4.5 kg with a history of two dead siblings presented with fit, hypoglycemia, hypocalcemia, and septicemia. Regarding the morbidity pattern in the study group, 36 cases (72%) had hypoglycemia, and the fewest (2 cases) had birth asphyxia (4%) (Table 1). We compared the mortality pattern between the study and control group (Figure 6). In addition, one case of developmental dysplasia of the hip (DDH) in the study group was diagnosed by ultrasound.

**Table 1:** The percent of morbidities for study and control group.

Type of morbidity	Study group n=50, No (%)	Control group n=50, No (%)	P-value	Odds ratio	C.I
Hypoglycemia	36 (72%)	4 (8%)	0.00*Sig.	29.5	8.9–97.5
Macrosomia	29 (58%)	6 (12%)	0.00005*Sig.	6.8	2.5–18.4
Neonatal jaundice (NNJ)	24 (48%)	13 (26%)	0.02*Sig.	2.6	1.1–6.1
Hypocalcemia	22 (44%)	3 (6%)	0.00001*Sig.	12.3	3.3–44.9
Transient tachypnea of the newborn (TTN)	21 (42%)	16 (32%)	0.3**N.S	1.5	0.6–3.4
Polycythemia	16 (32%)	2 (4%)	0.0002*Sig.	11.3	2.4–52.3
Respiratory Distress Syndrome (RDS)	13 (26%)	11 (22%)	0.6**N.S.	1.2	0.5–3.1
Prematurity	9 (18%)	9 (18%)	1**N.S	1	0.4–2.8
Coronary heart disease (CHD)	5 (10%)	1 (2%)	0.09**N.S	5.4	0.6–48.3
Birth injury	3 (6%)	0 (0%)	0.07**N.S	-	-
Birth asphyxia	2 (4%)	12 (24%)	0.003*Sig.	0.13	0.028–0.63 Protective

* Sig. – significant; **N.S – not significant.

**Figure 7:**
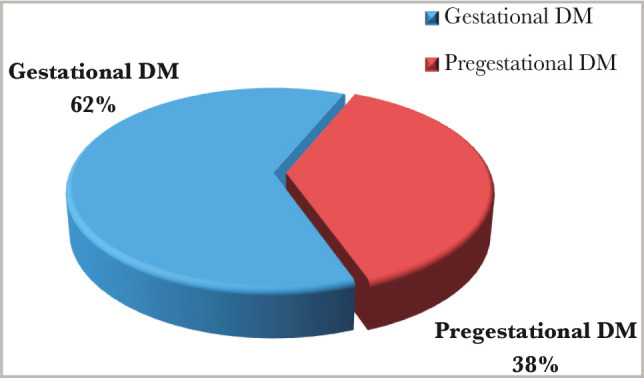
Type of diabetes among mothers in the study group.

Out of fifty patients in the study group, 31 (62%) were gestational diabetic mothers, and 19 (38%) were pre-gestational diabetic mothers, as shown in Figure 7.

Table 2 lists the distribution of morbidity between pregestational and gestational diabetes. Macrosomia, transient tachypnea of the newborn (TTN), birth injury, and birth asphyxia were more prevalent in gestational than pre-gestational diabetes, while metabolic disturbance and prematurity were more prevalent in pre-gestational diabetes.

**Table 2:** Morbidity patterns among patients of gestational DM and pregestational DM mothers.

Type of morbidity	PGD n=19, No (%)	GD n=31, No (%)	P-value	Odds ratio	C.I
Hypoglycemia	14 (73.6%)	22 (70.9%)	0.09**N.S	0.5	0.22–1.14
Macrosomia	9 (47.3%)	20 (64.5%)	0.02 *Sig.	0.3	0.13–0.82 Protective
NNJ	10 (52.6%)	14 (45.2%)	0.3**N.S	0.6	0.3–1.6
Hypocalcaemia	9 (47.4%)	13 (41.9%)	0.3**N.S	0.6	0.2–1.6
TTN	5 (26.3%)	16 (51.6%)	0.006*Sig.	0.24	0.08–0.7 Protective
Polycythemia	7 (36.8%)	9 (29%)	0.5**N.S	0.7	0.25–1.28
RDS	4 (21%)	9 (29%)	0.13**N.S	0.4	0.1–1.4
Prematurity	4 (21%)	5 (16%)	0.73**N.S	0.8	0.2–3.1
CHD	1 (5.3%)	4 (12.9%)	0.17**N.S	0.2	0.03–2.2
Birth injury	0 (0%)	3 (9.7%)	0.07**N.S	-	-
Birth asphyxias	0 (0%)	2 (6.5%)	0.2**N.S	-	-

* Sig. – significant; **N.S – not significant.

In the study group, most mothers had advanced maternal age (31 mothers >35 years), and all of them were multiparous, two mothers with a history of 2 dead siblings with hypoglycemia and RDS, and one mother with a history of 1 dead sibling with the same condition. Seven mothers had a history of a previous sibling with hypoglycemia and admission to the nursery care unit, and one mother had a history of a previous sibling with RDS, hypoglycemia, and admission to the nursery unit. Details regarding the treatment of mothers are described in Table 3. Two of three mothers had primary hypertension, and only one had pregnancy-induced hypertension. Most mothers with DM gestational or pregestational had high blood glucose levels even with treatment.

**Table 3:** Treatment of diabetic mothers.

Type of DM	Diet	Insulin	Oral hypoglycemic	Total
**Pre-gestational DM**	0	12	7	19
**Gestational DM**	12	10	9	31

## Discussion

In this study, our patients belonged to the neonatal age group (0 to 26 days), the majority below two weeks (n=50 study group & control group). The slight male predominance observed in this study with a ratio of male:female (1.8:1 in the study group and 1.2:1 in the control group) (Figures 1 and 2) agrees with another study [9].

Regarding the gestational age, 18% were preterm patients, and 82% were patients born at term, which agrees with another study [10]. However, there was no significant difference between the study and control group, most probably because we chose the control group according to the gestational age matching the study group. The caesarian section (C/S) rate in this study was exceptionally high (86% for the study group and 58% for the control group), with elective C/S rates being higher than emergency rates even amongst the controls. The high rate of cesarean section deliveries, similar to other studies [11], is associated with the high incidence of macrosomia in the IDMs, and maternal variables (previous scar, tubal ligation etc) in the control group. Emergency C/S rates are substantially greater than elective C/S rates [9]. This is because women frequently register late for antenatal care (ANC), and surgical deliveries are widely accepted. According to the findings, 13 (26%) diabetic mothers had previously experienced early newborn mortality and morbidities (as documented in their case notes). Diabetes plays an important role in prenatal and neonatal mortality. The study also found that IDMs have a lower death rate than non-IDMs, which is consistent with other studies [9, 10]. Although perinatal mortality decreased in this group, neonatal morbidity remains a serious concern. In this study, hypoglycemia was the most common newborn issue seen in IDMs, affecting considerably more infants than those born to non-diabetic mothers. The high rate of hypoglycemia in IDMs (72% for the study group and 8% for the control group with a significant P-value of 0.00 and C.I. of 8.9–97.5) is similar to findings of other studies [10, 11] and was identified as a marker of poor glycemic control in the mother. A faster decrease in plasma glucose concentration is an indicator of IDM, especially in those whose mother's diabetes is poorly controlled [12]. Maternal hyperglycemia usually causes fetal hyperglycemia, stimulating the fetal pancreas to synthesize excessive insulin. With placenta separation at birth, the glucose infusion to the neonate will suddenly stop without a proportional effect on hyperinsulinemia [13]. There was a high percentage of macrosomia among IDMs in this study, consistent with others [10, 11]. Macrosomia remains an important morbidity because it is associated with an increased risk for traumatic birth injury (6% in our study group and 0% in the control group), obesity, and diabetes in later life. Most authors agree that macrosomia is in part related to maternal glucose control [14]. Our IDMs had a high rate of neonatal jaundice (NNJ), 48% *vs*. 26% in the control group. The high levels of NNJ in our IDMs disagreed with another study [9], where it occurred in only 29% of cases. The reason for this high percentage is not clear. Hyperbilirubinemia is a common problem of IDM and has been shown to occur with increased frequency in macrosomic infants of diabetic mothers [15]. Hyperbilirubinemia occurred in most of the present macrosomic IDMs. However, the association was not statistically significant. The second most common metabolic disturbance in our study was hypocalcemia (44% in the study group *vs*. 6% in the control group), which is in line with Opara *et al*. [9]. A hypothesis was that hyperparathyroidism in diabetic mothers might suppress the fetal parathyroid gland function and lead to hypocalcemia in the newborn [16, 17]. The hematocrit was more than 65% present in 32% of the study group *vs*. 4% of the control group, which is not in line with Opara *et al*. [9] and Aysar *et al*. [11]. This study showed that 42% of patients had transient tachypnea of the newborn (TTN) in the study group *versus* 32% in the control group, and 26% of patients had respiratory distress syndrome (RDS) compared to 22% in the control group. Both TTN and RDS were high but not statistically significant, which is in line with Opara *et al*. for the TTN but not for RDS [9]. TTN is a known complication of IDMs, especially following C/S deliveries [18]. A high percentage of RDS recorded in this study agreed with other Iraqi studies [10, 11] because it occurs more frequently in IDMs than in normal infants [19].

The incidence of major congenital malformations was about 2–5 times greater in IDMs than in other infants, with cardiac malformations recorded as the most common. In our study group, the percentage of coronary heart disease (CHD) was 10% *versus* 2% in the control group, which is higher but statistically insignificant. There were no obvious congenital malformations in the current study, which agrees with another study [10], which looked at outcomes in pregnant women. However, we looked only at infants admitted to our unit, potentially excluding malformations that were not compatible with life. This could be because our study group only enrolled 50 patients, which is a small number. The birth injury in our study was higher in the study group, 6% *versus* 0% in the control group, but statistically insignificant. In contrast to the above, the statistics in birth asphyxia were significant for the control group, 24% *vs*. 4% in the study group, which may be due to the high rate of C/S among IDMs. We compared infants of gestational diabetes mellitus (GDM) and pre-gestational diabetes (PGDM) mothers regarding the morbidity pattern. The study did not show any significant difference in morbidity patterns except in macrosomia which was 47.3% in PGDM *versus* 64.7% in GDM. This may be due to the late detection of GDM and poor compliance to the treatment in our society, so macrosomia increased in infants of GDM mothers. Also, there was a significant difference in TTN, 26.3 in PGDM, *versus* 51.6% in GDM, which was not in line with Opara *et al*. and others [9, 10].

## Conclusion

Diabetes during pregnancy has a serious effect on neonates and their mothers. The commenced morbidities in IDMs were hypoglycemia, macrosomia, and hyperbilirubinemia, so strict control of blood glucose level during pregnancy and education of diabetic women is essential before and during gestation.
